# Vitamin D status in chimpanzees in human care: a Europe wide study

**DOI:** 10.1038/s41598-022-21211-6

**Published:** 2022-10-21

**Authors:** Sophie Moittié, Rachel Jarvis, Stephan Bandelow, Sarah Byrne, Phillipa Dobbs, Melissa Grant, Christopher Reeves, Kate White, Mátyás Liptovszky, Kerstin Baiker

**Affiliations:** 1 School of Veterinary Medicine, St. George’s University, West Indies, Grenada; 2 School of Veterinary Medicine and Science, University of Nottingham, Sutton Bonington, LE12 5RD UK; 3 School of Medicine, St. George’s University, West Indies, Grenada; 4 Twycross Zoo, Atherstone, CV9 3PX UK; 5 School of Dentistry, Institute of Clinical Science, University of Birmingham and Birmingham Community Healthcare Foundation Trust, Birmingham, B5 7ET UK; 6 Department of Clinical Biochemistry, Manchester University NHS Foundation Trust, Manchester, UK; 7 Department of Veterinary Clinical Sciences, City University of Hong Kong, Kowloon, Hong Kong SAR Hong Kong; 8 Present Address: Dublin Zoo, Saint James’, Dublin 8, Ireland; 9 Present Address: Department of Life Sciences, Perth Zoo, South Perth, WA 6151 Australia

**Keywords:** Biomarkers, Cardiology, Diseases, Health care, Pathogenesis, Risk factors

## Abstract

While vitamin D deficiency is a public health concern in humans, comparatively little is known about vitamin D levels in non-human primates. Vitamin D plays a crucial role in overall health and its deficiency is associated with a range of disorders, including cardiovascular disease, which is a leading cause of death in great apes. Serum samples (n = 245) from chimpanzees (*Pan troglodytes*) housed at 32 European zoos were measured for 25-hydroxyvitamin D_2_, 25-hydroxyvitamin D_3_ and total 25-hydroxyvitamin D (25-OHD) using liquid chromatography and tandem mass spectrometry. Of these samples, 33.1% indicated inadequate vitamin D status, using the human reference interval (25-OHD < 50 nmol/L). The season of the year, health status of the animal, and the provision of daily outdoor access had a significant effect on vitamin D status. This is the first large-scale study on vitamin D status of non-human great apes in human care. Inadequate 25-OHD serum concentrations are widespread in the chimpanzee population in Europe and could be a risk factor for the development of idiopathic myocardial fibrosis, a major cause of mortality in this species, as well as other diseases. A review of husbandry and nutrition practices is recommended to ensure optimal vitamin D supply for these endangered animals.

## Introduction

Vitamin D deficiency in humans is being described as a pandemic, and is associated with healthcare costs of billions of Euros in Europe^1,2^. Apart from the well-known role of vitamin D in calcium homeostasis and the musculoskeletal system, vitamin D has a wide range of other biological functions and prolonged vitamin D deficiency has been associated with a variety of disorders in humans such as cardiovascular diseases, neurological disorders, cancers, autoimmune diseases, and respiratory infections^3–6^.

Vitamin D comprises a range of fat-soluble secosteroids that are synthesised in the skin from ultraviolet B (UVB) radiation (vitamin D_3_) or obtained from dietary sources (vitamin D_2_ and D_3_)^4^. Both vitamin D_2_ and D_3_ are inactive forms that are converted in the liver into 25-hydroxyvitamin D_2_ (25-OHD_2_) and 25-hydroxyvitamin D_3_ (25-OHD_3_), the sum of which is referred to as total 25-hydroxyvitamin D (25-OHD). 25-OHD is the major circulating metabolite and the recommended biomarker used to measure vitamin D status due to its stability and long half-life^7,8^. 25-OHD is converted by the kidneys into the active form 1,25-hydroxyvitamin D, which interacts via the vitamin D receptor, a nuclear hormone receptor expressed in many cells including cardiomyocytes and is thought to directly regulate over 2000 genes^4,9^.

The effects of vitamin D are likely to be similar in great apes and humans due to their genetic and phenotypic similarities, thus vitamin D deficiency could have a severe negative impact on the health of great apes. The authors have previously hypothesised the role of vitamin D deficiency in ape cardiovascular disease (CVD), which is a major cause of morbidity and mortality in captive great apes^10,11^. One such CVD, idiopathic myocardial fibrosis (IMF), where cardiac muscle is progressively replaced by connective tissue, affects up to 91% of zoo-housed chimpanzees (*Pan troglodytes*) in Europe and 77% in the United States^11,12^. However, little has been published regarding the aetiopathogenesis and risk factors associated with myocardial fibrosis in chimpanzees^13,14^. Husbandry and nutrition need to be investigated as possible risk factors in great ape CVD, as research indicates that zoo-housed great apes are more affected by CVD than their wild counterparts^11^. An inhibitory role for vitamin D in cardiac fibrosis has been demonstrated in humans and animals^15–17^, principally through inhibition of the main pro-fibrotic factor transforming growth factor beta 1 (TGF-β1). There is also an inverse relationship between serum 25-OHD and parathyroid hormone (PTH) concentrations in humans, with progressively increasing PTH when 25-OHD falls under 75 nmol/L. It was previously demonstrated that increasing PTH levels increase blood pressure, cause cardiomyocyte hypertrophy, and interstitial fibrosis of the heart^18^. Inadequate vitamin D status could thus be a risk factor for the development of IMF in chimpanzees^11^.

Solar UV radiation exposure is significantly more effective at increasing serum 25-OHD than dietary vitamin D_2_ or D_3_ supplementation^19–21^. Vitamin D synthesis in the skin is affected by environmental and individual variables such as available UVB, exposure time, skin pigmentation, and age^21,22^. All wild non-human great apes, including chimpanzees, live in tropical areas that receive high amounts of direct sunlight. They are thus likely poorly adapted to restricted UVB exposure and may not synthesise enough cutaneous vitamin D when housed in zoos at more northern or southern latitudes than their natural range^23^. Melanin, the main skin pigment, has an important adaptive role in protecting against UVA damage and in regulating UVB absorption, thus decreasing cutaneous vitamin D_3_ synthesis^8,22,24^. Therefore, dark skin is a well-known risk factor in humans for vitamin D deficiency, especially at northern latitudes, due to the lower average solar UV radiation^24,25^. Skin pigmentation also had an effect on vitamin D status in wild baboons, in that darker-skinned species were shown to have lower levels of vitamin D_3_ metabolites than lighter species^26^. Though some natural variation occurs with age and sun exposure, chimpanzees commonly have dark skin pigmentation, which may affect their ability to synthesise vitamin D when living outside their natural distribution range^27^. Although reference intervals for serum 25-OHD have not yet been established in non-human great apes, some small-scale studies suggest that both juvenile and adult captive chimpanzees may suffer from vitamin D deficiencies despite vitamin D supplementation^28–31^. At one US institution, chimpanzees housed in indoor-only enclosures had significantly lower vitamin D levels than chimpanzees with daily outdoor access^28^.

While vitamin D may have significant health impacts in chimpanzees, there are no standardised methods for measuring vitamin D status in this species. Analytical variability is one of the main challenges in measuring vitamin D metabolites, as it has been demonstrated that inter-assay and inter- and intra-laboratory variability commonly exceeds recommended allowable error^32,33^. Studies on vitamin D status in the European human population show very different estimates of prevalence of vitamin D deficiency, partly due to differences in analytical methods, and although it has been suggested to use centralised laboratories and standardised methods to make these studies more reliable, this has not yet been achieved^1,34^. A recent method validation study measuring 25-OHD in chimpanzees also highlighted the significant analytical variability between assays and laboratories when measuring 25-OHD in great apes^35^. Using liquid chromatography and tandem mass spectrometry (LC–MS/MS), which is considered the gold-standard method in human laboratory practice, at a single laboratory is thus key to precisely assess the vitamin D status of the chimpanzee population in Europe^36^. The aims of the present study were the following:

1. Evaluate the vitamin D status of European zoo chimpanzees using LC–MS/MS on serum samples at a single accredited laboratory.
2. Assess the effect of environmental variables (latitude, UVB irradiance, season) on chimpanzee 25-OHD concentrations.
3. Assess the impact of individual variables (health status, skin tone, body condition score, age, sex) on chimpanzee 25-OHD concentrations.
4. Evaluate the influence of husbandry practices in European zoos (provision of daily outside access, diet and vitamin D supplementation) on chimpanzee 25-OHD concentrations.

## Methods

Serum samples were received from European zoos and sanctuaries, that agreed to participate in this study and had available banked serum samples. Samples were in all cases taken opportunistically while chimpanzees were under anaesthesia for procedures such as scheduled health-checks, disease investigation, surgery, or transport within or between facilities. Samples were kept frozen at − 20 °C to − 80 °C and protected from the light after sampling at their facility of origin or at the European Association of Zoos and Aquaria (EAZA) Biobank. All available samples were sent on ice to an accredited laboratory participating in the United Kingdom Accreditation Service (UKAS) Vitamin D External Quality Assessment Scheme (Clinical Biochemistry—Manchester University NHS Foundation Trust) for measurements of 25-OHD_2_, 25-OHD_3_ and total 25-OHD using Transcend II liquid chromatography. The analysis required a sample preparation system with TurboFlow online sample preparation technology and a TSQ Endura tandem quadrupole mass spectrometer (Thermo Fisher Scientific), as detailed elsewhere^35^. Known human reference intervals were used to interpret chimpanzee vitamin D status, where 25-OHD > 50 nmol/L is currently considered sufficient^21,37^.

The following epidemiological information about each animal was obtained from participating zoos: age, sex, health status, body condition score, skin tone, body hair coverage, diet including any commercial dry feed (referred to as pellets) and vitamin D supplementation given, and details on provision of outdoor access in the 2 months prior to sampling. The geographic location (latitude and longitude) of each zoo was determined using Google Maps. Northern Europe was defined as latitudes above 46°N, where levels of UVB are too low to allow cutaneous vitamin D_3_ synthesis for most of the year^23^. For each sample, the mean UVB irradiance in W/m^2^ for the corresponding zoo location during the 2 months before sampling was calculated using daily local irradiance data available from the National Aeronautics and Space Administration (NASA) Langley Research Center (LaRC) Prediction of Worldwide Energy Resource (POWER) Project (Data Access Viewer v2.0.0). The time of sample storage before analysis was calculated in months. Animals were classified as healthy when reported as such or when reported to suffer from acute traumatic injuries only. Details on the categorical variables and the ordinal level grouping used for statistical analysis are shown in Table 1.

**Table 1:** Categorical variables used for statistical analysis.

Variable	Type	Levels
Age group^38^	Ordinal	Juvenile: < 15 years
		Adult: 15–34 years
		Elderly: > 34 years
Sex	Binary	Male
		Female
Zoo latitude^23^	Binary	Northern Europe: > 46°N
		Southern Europe: ≤ 46°N
Season of sampling	Categorical	Summer: 21st of June–21st of September
		Autumn: 22nd of September–20th of December
		Winter: 21st of December–20th of March
		Spring: 21st of March–20th of June
Skin tone	Binary	Dark coloured face
		Light coloured face
Hair coverage	Ordinal	Good coat condition
		Partial hair loss
		Total hair loss
Provision of vitamin D in the form of oral supplementation (not including fortified pellets) at the time of sampling	Binary	No provision of extra vitamin D
		Provision of extra vitamin D
Provision of outside access in the 2 months before sampling	Binary	Limited outside access
		Unlimited outside access
Body condition score (BCS) reported on a scale of 1–9	Ordinal	Underweight (BCS 1–3)
		Optimal (BCS 4–6)
		Overweight (BCS 7–9)
Health status at the time of sampling	Binary	Healthy (including acute trauma)
		Abnormal health (any disease reported except acute trauma)

To account for the repeated measures of vitamin D levels within the same individuals, mixed effects models with a random effect for each individual were used. The analyses were performed with R version 4.1.1 and the lme4 package for mixed effects models, which were built with a restricted maximum likelihood (REML) criterion. There is no requirement for normality of the outcome variable for these models, normality of the model residuals was assessed to establish model fit. Package lmerTest was used for significance testing and degrees of freedom estimation in these models, using the conservative Satterthwaite method^39^. The significance cut-off was set at alpha = 0.05.

The project was under ethical review and was granted full ethical permission by The University of Nottingham, School of Veterinary Medicine and Science Ethics committee in adherence to institutional, national and international guidelines (ethics number 3064200106). Permission from each facility was given for investigation of every animal and no chimpanzees were euthanised or harmed for the purposes of research. Blood samples were taken in each zoo under the Veterinary Surgeons Act (VSA) or equivalent guidelines and regulations in their country. All samples were shipped with full CITES (Convention on International Trade in Endangered Species of Wild Fauna and Flora) export and import permits. Study methods were carried out in accordance with the ARRIVE guidelines.

## Results

A total of n = 245 serum samples were analysed, from 140 individual chimpanzees from 32 European zoos and sanctuaries. The number of samples per individual chimpanzee ranged from 1 to 10.

**Figure 1:**
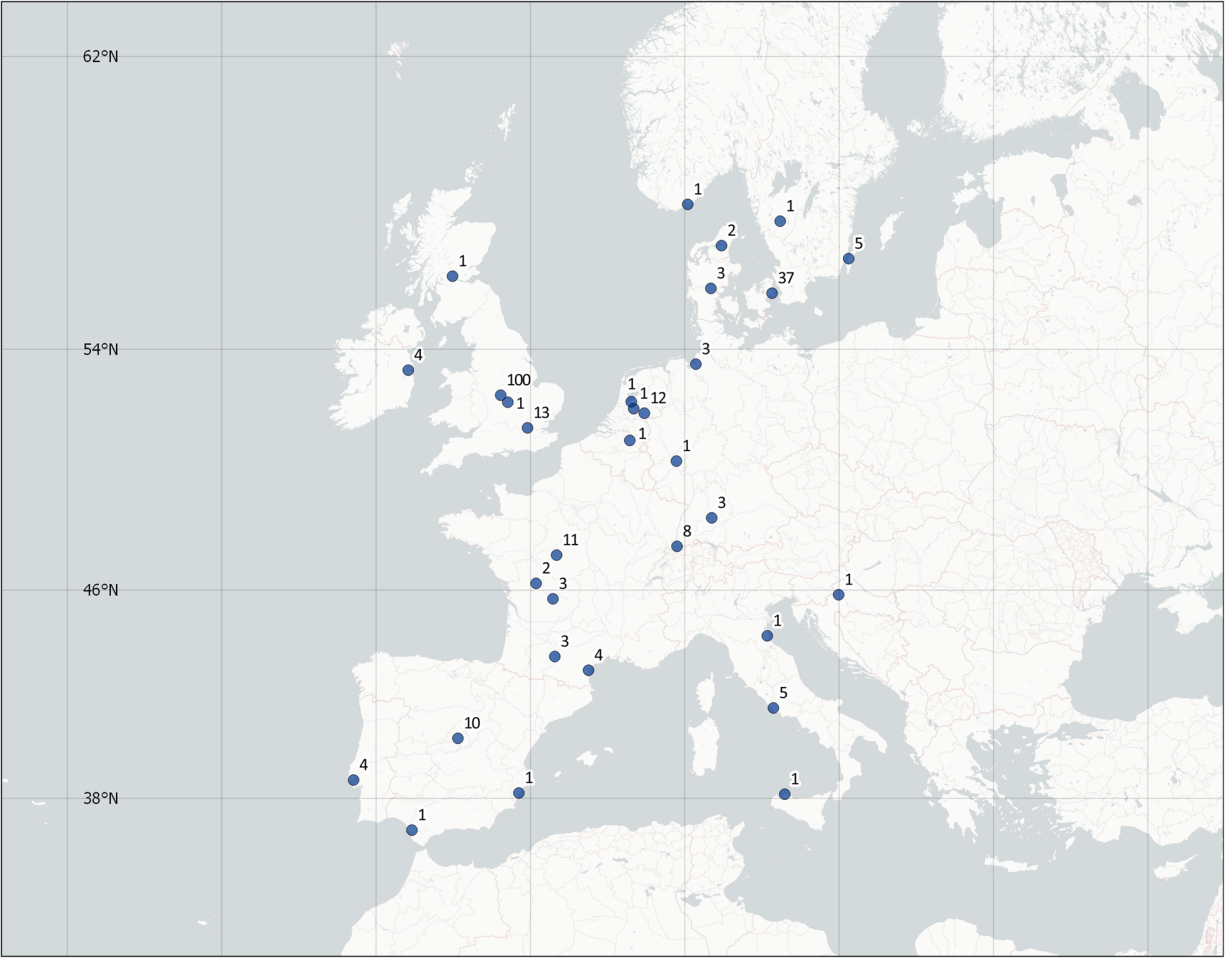
Geographic distribution and number of samples obtained from European zoos and sanctuaries for serum 25-OHD measurement. This map was created using QGIS Desktop v2.18.28.

The numbers of samples per facility ranged from 1 to 100. The largest number of samples was obtained from a British zoo (n = 100, latitude 52.65°). Figure 1 shows the number of samples submitted by European zoos and sanctuaries.

Chimpanzees’ median age at the time of sampling was 27 years (range 1–65 years), with 46 samples corresponding to juvenile individuals, 130 to adults and 65 to elderly.

A total of 97 samples were from male chimpanzees, and 147 from females. Reported health conditions of the chimpanzees were varied and included metabolic diseases like diabetes mellitus, neoplastic processes, chronic renal disease, cardiac, dental, and infectious diseases. A total of seven chimpanzees (corresponding to 11 samples) were known or suspected to suffer from cardiac disease at the time of sampling.

### General vitamin D status

The concentration of 25-OHD_2_ was less than 5 nmol/L thus negligible for all samples except for three: one chimpanzee from a British zoo with 25-OHD_3_ = 26.2 nmol/L and 25-OHD_2_ = 14.8 nmol/L, and two chimpanzees from a Danish zoo with concentrations of 25-OHD_3_/25-OHD_2_ = 23/15.7 nmol/L and 25.9/27.7 nmol/L respectively. Thus, apart from these 3 chimpanzees, the total 25-OHD results in this study were equivalent to the concentrations of 25-OHD_3_, thus total 25-OHD was used as the key outcome variable. One sample that had a total 25-OHD concentration below the detection limit of 5 nmol/L, this result was considered equal to 5 nmol/L for statistical analysis.

**Figure 2:**
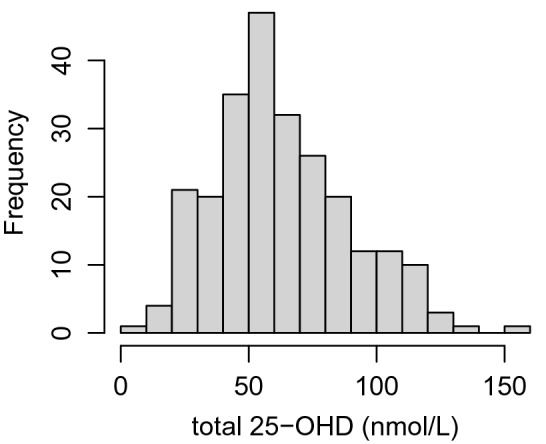
Frequency distribution of total 25-OHD in European zoo chimpanzees.

A histogram of 25-OHD concentrations showed some indication of skew (Fig. 2), but a log-transformation did not result in a more normal distribution of values. Accordingly, the uncorrected total 25-OHD levels were used as the outcome scores reflecting overall vitamin D status. 25-OHD concentrations ranged from < 5 to 151 nmol/L with a median of 57.7 nmol/L. A total of 81 samples (33.1%) had concentrations of 25-OHD below the human deficiency threshold of 50 nmol/L.

### Significant predictors of vitamin D levels

To build a robust model of vitamin D status, all individually significant predictor variables for total 25-OHD levels were entered into a mixed effects model with main effects only at the first step. UVB irradiance analysed continuously dropped out of significance (p = 0.07) when sampling season was added into the same model, indicating that the latter explains the variance in 25-OHD levels better than the 2-month UVB irradiance. To build the most robust model, UVB irradiance scores were removed from the global model and analysed separately instead (see section on UVB irradiance below).

**Figure 3:**
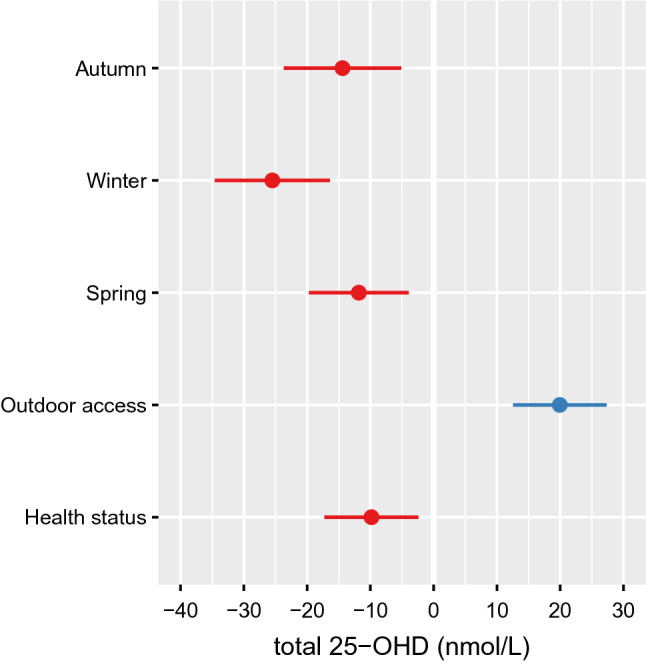
Effect size estimates and 95% confidence intervals for the main effects of all significant predictors of total 25-OHD levels. Summer 25-OHD levels are used as the baseline reference.

The model then contained only significant main effects. Interactions were also included where theoretically plausible but did not improve model fit in any case. The final model was thus restricted to only include significant main effects, which yielded a good fit with a conditional coefficient of determination (adjusted R^2^) of 53%. The distribution of residuals resembled a normal distribution very closely, and Q-Q plots also indicated good model fit. The model results are listed in Table 2 and illustrated in Fig. 3. Summer 25-OHD levels were used as the baseline reference for seasonal effects. Compared to the summer season, 25-OHD concentrations were 14.4 nmol/L lower in autumn, 25.5 nmol/L lower in winter, and 11.8 nmol/L lower in spring. Unlimited outdoor access increased 25-OHD levels by 19.9 nmol/L whereas poor health status was associated with a 9.8 nmol/L decrease in 25-OHD levels.

**Table 2:** Effect sizes and significance estimates for all significant predictors of total 25-OHD levels.

Predictor	Effect size (nmol/L)	df	t	p
Unlimited outdoor access	19.9	113	5.32	5.35e−07
Season: autumn	− 14.4	152	− 3.06	0.0026
Season: winter	− 25.5	146	5.53	1.43e−07
Season: spring	− 11.8	148	− 2.96	0.036
Health status	− 9.8	165	− 2.61	0.0098

### Seasonal effects

**Figure 4:**
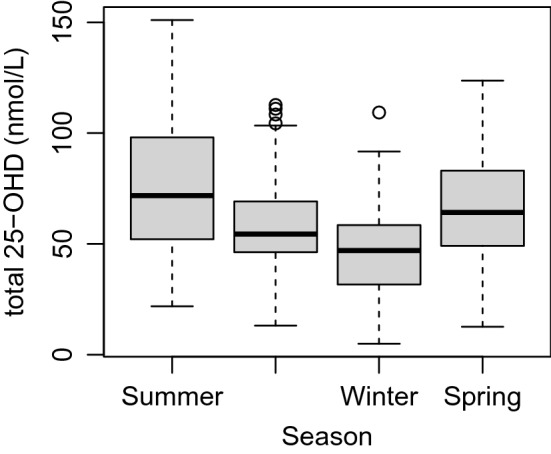
Total 25-OHD by season in European zoo chimpanzees. Central black bar: median. Inner box: interquartile range. Whiskers: range without outliers. Circles: outliers.

Because the global model showed a significant effect of sampling season on vitamin D levels, uncorrected seasonal levels are presented in more detail in this section. Sampling date was known for 244 samples. 49 samples were taken in winter, 100 in spring, 47 in summer, and 48 in autumn. Median 25-OHD concentrations were 47 nmol/L in winter (IQR = 31.2–59.5 nmol/L), 64.20 nmol/L in spring (IQR = 49–83.1), 71.8 in summer (IQR = 51.1–100.4) and 54.5 in autumn (IQR = 46.2–69.5) (Fig. 4). The percentage of samples that were below the human deficiency threshold of 50 nmol/L per season were: 55.1% in winter, 28% in spring, 23.4% in summer, and 31.3% in autumn.

### UVB irradiance

**Figure 5:**
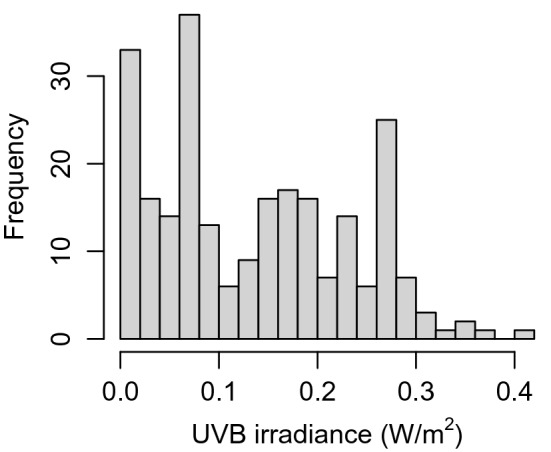
Distribution of UVB irradiance means in the 2 months before taking the sample. Bin size = 0.02.

**Figure 6:**
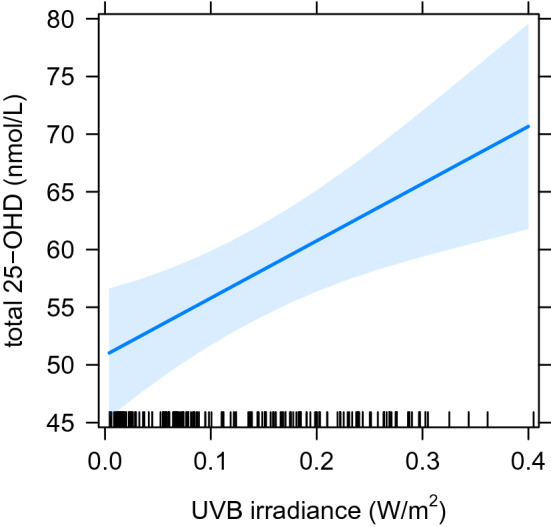
Estimates for the effect of UVB irradiance on 25-OHD levels, showing regression line and 95% confidence interval (shaded band).

Mean UVB irradiance in the 2 months before sampling was analysed separately as a continuous variable. As expected, the distribution also exhibited some right-hand skew (Fig. 5), but again a log transformation did not lead to a more symmetrical distribution. The uncorrected UVB irradiance scores were thus used for further analysis. Although sampling season appears to be a more robust predictor of 25-OHD levels than UVB irradiance in the 2 months prior to taking the sample, the continuous UVB irradiance scores are a significant predictor of 25-OHD levels when analysed in isolation (effect size = 49.6 nmol/L per 1 W/m^2^, t(1, 232) = 3.24, p = 0.0014, see Fig. 6). The continuous UVB irradiance was then used to determine if there is a significant non-linear relationship in the effect on 25-OHD levels, which might indicate saturation at higher levels of UVB radiation. However, the non-linear effect was not significant (p = 0.42), which implies that there was no saturation effect for UVB radiation.

### Variables that did not predict vitamin D levels

All other variables were also tested for a significant main effect on 25-OHD levels in separate mixed effects models containing no other co-variates. They did not exhibit significant effects, also when included in the more complex main model. Their main effect p-values and total number of valid data points are listed in Table 3 for reference.

**Table 3:** Non-significant predictors of 25-OHD levels.

Variable	Valid n	p
Sample storage (months)	244	0.147
Sex	244	0.732
Latitude (continuous)	245	0.647
Latitude (binary North/South)	245	0.245
Skin tone	181	0.463
Hair coverage	168	0.491
Vitamin D supplementation	162	0.964
BCS	187	0.288
Age category	241	0.384

### Diet and supplementation

Information on the chimpanzees’ diet at the time of sampling was available for 196 (80%) samples. For 187 (95.4%) of these samples, the animals were reported to eat primate pellets as part of their diet at the time of sampling. Diets were largely based on green and root vegetables, pellet, fruits, nuts and occasional other food items, such as eggs, vegetable oil, or meat. The total and relative amount eaten by each individual animal was not possible to ascertain, as diets were reported for whole chimpanzee groups.

Although at least seven different types of primate pellets were identified as offered to animals included in this study, but many facilities were unable to ascertain exactly which type of pellets the animals were receiving at the time of sampling. The amount of vitamin D present in commercial pellets varied greatly depending on the type, ranging from 1600 to 10,170 IU/kg. One food distributor reported to have markedly increased the vitamin D amount in their pellets in 2018 from 3500 to 8500 IU/kg. It was not possible to statistically assess the effect of pellet type on serum 25-OHD concentrations, due to the limited number of samples for each type of pellet and the uncertainty on which and how much pellet was consumed by the animal at the time of sampling.

Only 21 animals (from seven different zoos) were reported to receive extra oral vitamin D supplementation in addition of the vitamin D present in the rest of their diet at the time of sampling (n = 23 samples). It was, however, difficult to ascertain the exact amount of vitamin D received by each animal due to the zoos mixing the supplementation with the group diet, or due to lack of detail on the exact vitamin D amount present in the supplementation. The provision of extra vitamin D supplementation did not appear to have an effect on serum 25-OHD concentrations (see Table 2).

## Discussion

This study represents the largest survey on vitamin D status of non-human great apes under human care. A significant strength in the methods used was the reduction in analytical variability by analysing all samples at the same laboratory with the same gold-standard assay, thus allowing a more accurate comparison between results^7^. Although pre-analytical factors such as storage and shipment conditions were expected to be different for all samples, the high stability of 25-OHD in serum samples, even if transiently kept at room temperature, justifies our choice of study methods^40,41^.

Though a number of studies have measured vitamin D status in non-human primates, reference intervals for 25-OHD in apes have not yet been established. Juvenile chimpanzees with serum 25-OHD of 4 to 32 nmol/L were diagnosed with dietary rickets, while in the same group apparently healthy adult chimpanzees had average 25-OHD levels of 31 nmol/L. Interestingly, when some of these chimpanzees were later moved to a facility with year-round sun exposure in Hawaii, their average 25-OHD levels raised to 97.3 nmol/L. Another study suggests that adult chimpanzees housed in indoor-only enclosures experience vitamin D deficiency, with 25-OHD levels averaging 38.2 nmol/L despite dietary supplementation^28,29^. When vitamin D status was assessed in nine species of zoo-housed primates, chimpanzees were found to have the lowest average 25-OHD serum levels (32.7 nmol/L) despite dietary levels above recommendations^30^. In zoo-housed Western lowland gorillas (*Gorilla gorilla gorilla*), serum 25-OHD levels were found to be low despite oral supplementation, with 25-OHD concentrations averaging 35.5 nmol/L in animals managed indoors and 66.6 nmol/L in animals with unrestricted outdoor access^42^. A study on the vitamin D status of baboons showed that sun exposed and supplemented olive baboons (*Papio anubis*) housed outdoor in Texas (USA) had average 25-OHD_3_ levels of 220 nmol/L, and that average 25-OHD_3_ levels for wild baboons were between 149.5 and 315.5 nmol/L depending on the species^26^. Reported levels of serum vitamin D metabolites in non-human primates differ widely depending on species, with New-World monkeys such as marmosets having significantly higher levels than other species (serum 25-OHD_3_ of supplemented zoo marmosets are often above 500 nmol/L)^43^. Although these studies give an insight about vitamin D status of captive and wild primates, cautious interpretation is warranted and comparison between studies is problematic, due to the relatively small number of samples analysed, different species studied and the use of heterogeneous methods.

For humans, the suggested definition of vitamin D deficiency varies depending on sources, but serum 25-OHD concentrations between 50 and 125 nmol/L are generally recommended, while levels below 30 nmol/L are considered severely deficient and can increase the risk of developing diseases such as rickets or osteomalacia^44–46^. Applying a conservative 50 nmol/L cut-off for chimpanzees, more than 33% of all samples collected in the present study could be considered deficient, with the proportion rising to 55.1% in winter. Zoo latitude was not a significant predictor of 25-OHD levels in chimpanzees, and 13 out of 81 samples (16%) with 25-OHD below 50 nmol/L were from chimpanzees living in Southern European facilities. This means that the whole European chimpanzee population could be at risk of increased morbidity and mortality due to a suboptimal vitamin D status.

There was a clear difference in 25-OHD concentration between seasons, with higher levels observed in summer while lower levels were observed in winter. It seems, however, that for a large proportion of chimpanzees, even at the end of summer serum 25-OHD concentrations may not be high enough to ensure that they will not become deficient by winter. In humans in the United Kingdom (UK), 25-OHD serum levels in September needs to be at least 80.5 nmol/L in order to exceed a concentration of 25 nmol/L in February^47^. Unsurprisingly, mean UVB irradiance the two months before sampling was also significantly associated with 25-OHD levels.

Of interest is the lack of significant plateau in the relationship between UVB radiation and 25-OHD levels, which suggests that maximal cutaneous vitamin D synthesis is not reached within the sampled range of UVB levels. Accordingly, more exposure to UVB radiation should result in higher 25-OHD levels in the entire chimpanzee population, which may yield additional health benefits even for individuals above the deficiency cut-off for humans. Average annual UVB irradiance in chimpanzee natural range areas is between 0.3–0.4 W/m^2^ (NASA LaRC POWER Project, Data Access Viewer v2.0.0), however in this study only eight samples were available where individuals were exposed to these levels. This highlights that solar UVB irradiance in Europe may be inadequate compared with levels encountered in the natural range of the species, further emphasising the need to review husbandry and nutrition practices for non-human great apes in human care.

The provision of unlimited daily outdoor access was associated with higher 25-OHD concentrations year-round. This is in accordance with the results of other studies on zoo gorillas and chimpanzees^28,42^.Although the provision of outdoor access during the majority of the year is recommended by the North American Association of Zoos and Aquariums’ Chimpanzee Species Survival Plan^48^, no recommendations have been published by the EAZA, and outdoor access is often restricted for chimpanzees in some zoos and sanctuaries for climatic, husbandry, or other animal management reasons. It is thus necessary to raise awareness about the importance of encouraging animals to spend time outdoors, especially during the high-UV season when cutaneous vitamin D synthesis is possible. In Caucasian humans in the UK, only nine minutes of daily sunshine exposure (with about one third of the total skin area exposed) during the high-UV season have been proposed to maintain 25-OHD levels above 25 nmol/L throughout winter^47^. Longer exposure times are likely to be necessary in chimpanzees to maintain healthy 25-OHD concentrations year-round, due to their darker skin and more extensive hair coverage. It may be safer to recommend daily sun or UVB exposure for zoo-housed chimpanzees, rather than advocate for vitamin D supplementation in the diet, as hypervitaminosis D can occur with supplementation, but not with UVB exposure^34,49^. Moreover, conflictive evidence exists from human studies regarding the impact of oral vitamin D supplementation on health outcomes^46,50^. And although sun-exposed captive baboons receiving oral supplementation had circulating 25-OHD levels comparable to wild baboons, cholecalciferol (vitamin D_3_) conversion to 25-OHD_3_ was higher in wild baboons compared to captive baboons receiving oral supplementation, supporting the assumption that UV exposure is more efficient than supplementation at raising circulating 25-OHD levels in non-human primate species^26^. Artificial UVB lighting is widely used in certain nondomestic taxa in human care, especially in reptiles and some small non-human primates (Callitrichids), but currently is not commonly utilised for non-human great apes due to cost, safety and efficacy considerations. This study provides some further evidence that investigating artificial UVB lighting as part of standard husbandry practices for non-human great apes at locations where available UVB levels are under the natural range (0.3–0.4 W/m^2^) might be of benefit, justifying the above-mentioned costs and difficulties.

There appeared to be a relationship between 25-OHD levels and health status in chimpanzees. Reported health conditions were diverse and clinical information was transmitted with different levels of details depending on the institution. The effect of vitamin D status on cardiac disease as such could not been statistically explored due to the relatively small number of chimpanzees known to suffer from cardiac disease at the time of sampling. This is not overly surprising as the ante-mortem diagnosis of CVD in chimpanzees is challenging, with the only definitive diagnostic method for chronic degenerative heart diseases like the commonly diagnosed IMF being post-mortem by cardiac histopathology. Chimpanzees with an abnormal health status in our study showed a significantly lower serum 25-OHD level than their healthy counterparts, but future studies will need to investigate this correlation further. In humans, vitamin D deficiency can not only lead to ill-health, but the inverse relationship is also observed, as chronic and acute inflammation can lead to a decrease in 25-OHD levels^44,51^.

Interestingly, age was not found to have significant effect on chimpanzee vitamin D status. In humans, prevalence of vitamin D deficiency has been shown to vary depending on age group in some, but not all, large-scale studies, with teenagers and the elderly often considered most at risk^1,52,53^. In wild baboons, juveniles were found to have higher vitamin D_3_ (cholecalciferol) levels, but not 25-OHD_3_ levels, than adults^26^. Other primate studies have shown an absent or weak effect of age on levels of vitamin D metabolites^28,42,43,54^.

The lack of a significant effect for variables with a smaller valid sample size, for which the analysis has reduced statistical power, is less likely to imply the confirmed absence of an effect. For example, analysing more samples from chimpanzees receiving extra vitamin D supplementation may have resulted in a significant effect of oral vitamin D supplementation on chimpanzee 25-OHD levels. However, the main limitations of this study were the relatively low number of samples from Southern Europe and the overrepresentation of samples from a single British zoo which likely led to bias when analysing the data. To have a clearer picture of vitamin D status in chimpanzees in Europe, ideally a wider and more balanced representation of latitudes would be needed, with a similar number of samples from Northern and Southern European zoos. This may however be difficult to achieve, as only one fifth of the European zoo chimpanzee population live in Southern Europe (according to Species360 Zoological Information Management System). That said, this study included samples from approximately 20% of the chimpanzee population kept in European zoos, which is an exceptional achievement considering that, due to the dangerous nature of these animals, samples are usually only taken opportunistically when chimpanzees are under general anaesthesia for health-checks, surgical procedures or enclosure moves.

Another limitation of our study is the inconsistency in information received from participating institutions about chimpanzee husbandry and nutrition at the time of sampling. Some institutions were not able to provide some or all of this information, which affected data analysis. This is common in large scale studies with numerous participating institutions, especially with archived data where some records may be incomplete or lost.

Hypovitaminosis D is a public health concern in humans and it has been linked to numerous chronic conditions including cancer, autoimmune, metabolic, and cardiovascular diseases, and a higher risk of overall mortality^55^. Growing evidence suggests that low vitamin D levels are associated with cardiovascular diseases such as hypertension, coronary heart disease, stroke, myocardial infarction, and cardiac fibrosis^15,16,56,57^. It is possible that vitamin D deficiency is a significant risk factor for cardiac disease in zoo-housed chimpanzees as macrophage-mediated fibrosis has been shown to induce differentiation of cardiac stromal cells into myofibroblasts^58–60^. A fibrosis-associated increase of macrophages and myofibroblasts was also shown in a previous study in chimpanzees^11^. Considering the results of this study, together with the high prevalence of idiopathic myocardial fibrosis (IMF) in chimpanzees^11^, a relationship between vitamin D status and cardiovascular disease seems plausible. Transforming growth factor beta1 (TGFβ1) is the principal pro-fibrotic factor and its selective inhibition or of one of its downstream effectors like Interleukin 11 could be a promising therapeutic target in cardiac fibrosis in humans and apes and needs to be further investigated^61,62^. Furthermore, models in rats and mice have shown that activation of the vitamin D receptor (VDR) can prevent adverse cardiac remodelling by fibrosis^63,64^. Clinical trials using a TGFβ inhibitor, increased UVB exposure and/or vitamin D supplementation could potentially represent an important treatment strategy not only for great apes in our care but also be a very valuable and suitable treatment option for humans with increased cardiac, renal, pulmonary, or hepatic fibrosis. Beyond cardiovascular diseases, vitamin D insufficiency could result in non-human great apes being more susceptible to bacterial or viral upper respiratory tract infections or pneumonia which are a common cause of morbidity in these species^65–67^.

Our study shows that a considerable proportion of European zoo-housed chimpanzees can be considered vitamin D deficient according to human reference levels. Health status and provision of outside access had significant impact on vitamin D status. Whether zoos should provide vitamin D supplementation cannot be decided based on this study, but similarly to humans, it may be advisable when sufficient cutaneous vitamin D synthesis is not possible. The provision of unlimited outdoor access, as well as regular health screenings including the measurement of serum 25-OHD concentrations are recommended to safeguard the health and welfare of the non-human great ape populations in human care.

## Data Availability

The datasets generated during and/or analysed during the current study are available from the corresponding author on reasonable request.
